# (*S*)-3-Acetyl-3-[(*R*)-1-(4-bromo­phen­yl)-2-nitro­eth­yl]oxolan-2-one

**DOI:** 10.1107/S1600536811051725

**Published:** 2011-12-14

**Authors:** Yifeng Wang, Ruxiang Chen, Zhaobo Li, Danqian Xu

**Affiliations:** a Catalytic Hydrogenation Research Center, Zhejiang University of Technology, Hangzhou 310014, People’s Republic of China; b Hangzhou Minsheng Pharmaceutical Group Co. Ltd, Hangzhou, People’s Republic of China

## Abstract

The title compound, C_14_H_14_BrNO_5_, has two chiral C atoms. The quaternary C atom in the oxolanone ring has an *S* configuration, while the adjacent tertiary C atom has an *R* configuration. The oxolanone ring adopts an envelope conformation, with the flap C atom lying 0.298 (3) Å from the mean plane of the remaining four atoms. In the crystal, mol­ecules are connected into chains along [010] *via* weak C—H⋯O hydrogen bonds.

## Related literature

For general background, see: Li *et al.* (2009 ▶), Malerich *et al.* (2008 ▶); For related structures, see: Li *et al.* (2005 ▶).
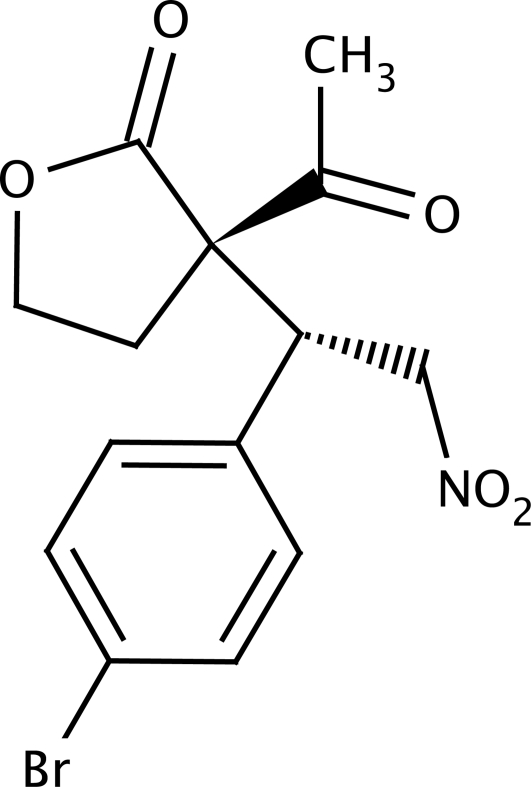


## Experimental

### 

#### Crystal data

C_14_H_14_BrNO_5_
*M* *_r_* = 356.17
Monoclinic, 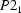

*a* = 9.6237 (7) Å
*b* = 6.6547 (4) Å
*c* = 12.0503 (8) Å
β = 105.794 (2)°
*V* = 742.60 (9) Å^3^
*Z* = 2
Mo *K*α radiation
μ = 2.79 mm^−1^
*T* = 296 K
0.43 × 0.27 × 0.22 mm

#### Data collection

Rigaku R-AXIS RAPID/ZJUG diffractometer
Absorption correction: multi-scan (*ABSCOR*; Higashi, 1995 ▶) *T*_min_ = 0.301, *T*_max_ = 0.542
7336 measured reflections
3272 independent reflections
1893 reflections with *I* > 2σ(*I*)
*R*_int_ = 0.041

#### Refinement

*R*[*F* ^2^ > 2σ(*F* ^2^)] = 0.033
*wR*(*F* ^2^) = 0.104
*S* = 1.00
3272 reflections
192 parameters
1 restraint
H-atom parameters constrained
Δρ_max_ = 0.45 e Å^−3^
Δρ_min_ = −0.45 e Å^−3^
Absolute structure: Flack (1983 ▶), 1327 Friedel pairs
Flack parameter: 0.014 (14)

### 

Data collection: *PROCESS-AUTO* (Rigaku, 2006 ▶); cell refinement: *PROCESS-AUTO*; data reduction: *CrystalStructure* (Rigaku, 2007 ▶); program(s) used to solve structure: *SHELXS97* (Sheldrick, 2008 ▶); program(s) used to refine structure: *SHELXL97* (Sheldrick, 2008 ▶); molecular graphics: *ORTEP-3 for Windows* (Farrugia, 1997 ▶); software used to prepare material for publication: *WinGX* (Farrugia, 1999 ▶).

## Supplementary Material

Crystal structure: contains datablock(s) global, I. DOI: 10.1107/S1600536811051725/pk2367sup1.cif

Structure factors: contains datablock(s) I. DOI: 10.1107/S1600536811051725/pk2367Isup2.hkl

Supplementary material file. DOI: 10.1107/S1600536811051725/pk2367Isup3.cml

Additional supplementary materials:  crystallographic information; 3D view; checkCIF report

## Figures and Tables

**Table 1:** Hydrogen-bond geometry (Å, °)

*D*—H⋯*A*	*D*—H	H⋯*A*	*D*⋯*A*	*D*—H⋯*A*
C11—H11⋯O5^i^	0.93	2.58	3.306 (2)	135
C10—H10⋯O3^ii^	0.93	2.63	3.514 (2)	158

Symmetry codes: (i) 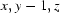; (ii) 
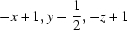
.

**Fig. 1.**
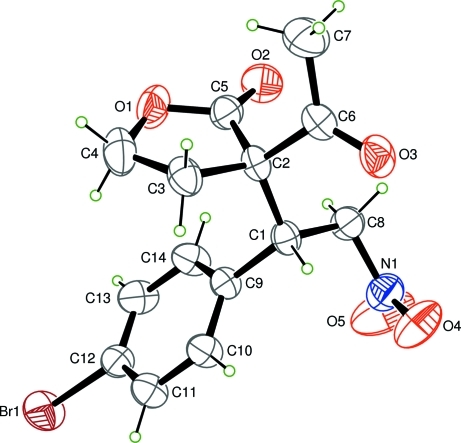
The asymmetric unit of the structure of the title compound. Displacement ellipsoids are drawn at the 50% probability level.

**Fig. 2.**
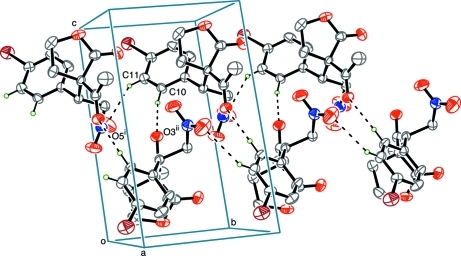
A view showing infinite chains with C11—H11···O5i and C10—H10···O3ii hydrogen bonds [Symmetry code: (i) x, -1 + y, z; (ii) 1 - x, -1/2 + y, 1 - z].

## supplementary crystallographic information

### Comment

The organocatalytic Michael reaction is often regarded as one of the most efficient and broadly applicable carbon-carbon bond-forming reactions. One of the most studied reactions is the Michael addition of 1,3-dicarbonyl compounds to nitroolefins because the highly functionalized nitro compounds are versatile intermediates in organic synthesis. The title compound, which was readily synthesized by the  organocatalytic Michael reaction of 3-acetyldihydrofuran-2(3*H*)-one to (*E*)-1-bromo-4-(2-nitrovinyl)benzene, could act as an intermediate in organic and natural product synthesis. In this article, the crystal structure of the title compoud (*S*)-3-acetyl-3-((*R*) -1-(4-bromophenyl)-2-nitroethyl)dihydrofuran-2(3*H*)-one is described (Fig. 1). The structure has two chiral centers. The quaternary carbon in the oxolanone ring of the title compound adopts an *S* configuration, while the adjacent tertiary carbon atom has *R* configuration.  The oxolanone ring displays an envelope conformation, with the flap carbon atom lying 0.298 (3) Å from the mean plane of the remaining four atoms. In the crystal, molecules are connected into chains along the *b* axis direction by weak C11—H11···O5^i^ and C10—H10···O3^ii^ hydrogen bonds [Symmetry code: (i)*x*, -1 + *y*, *z*; (ii) 1 - *x*, -1/2 + *y*, 1 - *z*] (Fig. 2).

### Experimental

To a solution of (*E*)-1-bromo-4-(2-nitrovinyl)benzene (1 mmol) and 3-acetyldihydrofuran-2(3*H*)-one (1 mmol) in 1,4-dioxane (3 ml) was added 3-((1*S*)-(6-methoxyquinolin-4-yl) (8-vinylquinuclidin-2-yl)methylamino)-4- ((*S*)-1-phenylethylamino)cyclobut -3-ene-1,2-dione(0.025 mmol) as catalyst, and the mixture was stirred at room temperature for 12 h (monitored by TLC). Then the solvent was distilled under vacuum, and the residue was purified by flash column chromatography (silica gel, Hex/AcOEt, *v*/*v*, 3:1) giving the title compound. Single crystals were obtained by slow evaporation of a CH_2_Cl_2_ and *i*PrOH solution.

### Refinement

H atoms were placed in calculated positions with C—H = 0.98 (1) Å (*sp*), C—H = 0.97 (1) Å (sp2), C—H = 0.96 (1) Å (sp3), C—H = 0.93 (1) Å (aromatic).  All H atoms included in the final cycles of refinement using a riding model, with *U*_iso_(H) = 1.2*U*_eq_ or 1.5*U*_eq_ (sp3) of the carrier atoms.

### Crystal data

**Table:**

C_14_H_14_BrNO_5_	*F*(000) = 360
*M**_r_* = 356.17	*D*_x_ = 1.593 Mg m^−^^3^
Monoclinic, *P*2_1_	Mo *K*α radiation, λ = 0.71073 Å
Hall symbol: P 2yb	Cell parameters from 5006 reflections
*a* = 9.6237 (7) Å	θ = 3.1–27.4°
*b* = 6.6547 (4) Å	µ = 2.79 mm^−^^1^
*c* = 12.0503 (8) Å	*T* = 296 K
β = 105.794 (2)°	Block, colourless
*V* = 742.60 (9) Å^3^	0.43 × 0.27 × 0.22 mm
*Z* = 2	

### Data collection

**Table:**

Rigaku R-AXIS RAPID/ZJUG diffractometer	3272 independent reflections
Radiation source: rotating anode	1893 reflections with *I* > 2σ(*I*)
graphite	*R*_int_ = 0.041
Detector resolution: 10.00 pixels mm^-1^	θ_max_ = 27.4°, θ_min_ = 3.2°
ω scans	*h* = −12→12
Absorption correction: multi-scan (*ABSCOR*; Higashi, 1995)	*k* = −8→8
*T*_min_ = 0.301, *T*_max_ = 0.542	*l* = −15→15
7336 measured reflections	

### Refinement

**Table:**

Refinement on *F*^2^	Hydrogen site location: inferred from neighbouring sites
Least-squares matrix: full	H-atom parameters constrained
*R*[*F*^2^ > 2σ(*F*^2^)] = 0.033	*w* = 1/[σ^2^(*F*_o_^2^) + (0.0212*P*)^2^ + 0.6532*P*] where *P* = (*F*_o_^2^ + 2*F*_c_^2^)/3
*wR*(*F*^2^) = 0.104	(Δ/σ)_max_ < 0.001
*S* = 1.00	Δρ_max_ = 0.45 e Å^−^^3^
3272 reflections	Δρ_min_ = −0.45 e Å^−^^3^
192 parameters	Extinction correction: *SHELXL97* (Sheldrick, 2008), Fc^*^=kFc[1+0.001xFc^2^λ^3^/sin(2θ)]^-1/4^
1 restraint	Extinction coefficient: 0.013 (2)
Primary atom site location: structure-invariant direct methods	Absolute structure: Flack (1983), 1327 Friedel pairs
Secondary atom site location: difference Fourier map	Flack parameter: 0.014 (14)

### Special details

**Table:**

Geometry. All e.s.d.'s (except the e.s.d. in the dihedral angle between two l.s. planes) are estimated using the full covariance matrix. The cell e.s.d.'s are taken into account individually in the estimation of e.s.d.'s in distances, angles and torsion angles; correlations between e.s.d.'s in cell parameters are only used when they are defined by crystal symmetry. An approximate (isotropic) treatment of cell e.s.d.'s is used for estimating e.s.d.'s involving l.s. planes.
Refinement. Refinement of *F*^2^ against ALL reflections. The weighted *R*-factor *wR* and goodness of fit *S* are based on *F*^2^, conventional *R*-factors *R* are based on *F*, with *F* set to zero for negative *F*^2^. The threshold expression of *F*^2^ > σ(*F*^2^) is used only for calculating *R*-factors(gt) *etc*. and is not relevant to the choice of reflections for refinement. *R*-factors based on *F*^2^ are statistically about twice as large as those based on *F*, and *R*- factors based on ALL data will be even larger.

### Fractional atomic coordinates and isotropic or equivalent isotropic displacement parameters (Å^2^)

**Table:**

	*x*	*y*	*z*	*U*_iso_*/*U*_eq_	
Br1	0.01386 (6)	0.39571 (12)	0.91217 (5)	0.0776 (3)	
O1	0.6151 (4)	0.8445 (6)	0.9285 (3)	0.0692 (12)	
O2	0.5776 (5)	1.1368 (6)	0.8363 (4)	0.0713 (12)	
O3	0.6176 (3)	0.9028 (8)	0.5481 (3)	0.0608 (8)	
O4	0.2157 (5)	0.9380 (9)	0.4296 (4)	0.0967 (17)	
O5	0.1045 (5)	1.0968 (9)	0.5321 (5)	0.121 (2)	
N1	0.2123 (6)	1.0229 (7)	0.5175 (4)	0.0647 (12)	
C1	0.4009 (5)	0.8284 (6)	0.6525 (4)	0.0429 (11)	
H1	0.3954	0.7478	0.5833	0.051*	
C2	0.5624 (5)	0.8272 (6)	0.7251 (4)	0.0424 (11)	
C3	0.6125 (6)	0.6151 (8)	0.7740 (5)	0.0550 (13)	
H3A	0.5456	0.5124	0.7346	0.066*	
H3B	0.7080	0.5846	0.7665	0.066*	
C4	0.6136 (8)	0.6296 (10)	0.9003 (5)	0.0751 (18)	
H4A	0.5284	0.5655	0.9125	0.090*	
H4B	0.6985	0.5636	0.9487	0.090*	
C5	0.5860 (5)	0.9572 (8)	0.8336 (4)	0.0524 (14)	
C6	0.6619 (5)	0.8984 (11)	0.6521 (4)	0.0505 (10)	
C7	0.8152 (6)	0.9490 (11)	0.7133 (5)	0.085 (2)	
H7A	0.8786	0.8842	0.6752	0.127*	
H7B	0.8368	0.9035	0.7917	0.127*	
H7C	0.8286	1.0919	0.7121	0.127*	
C8	0.3494 (6)	1.0384 (8)	0.6113 (4)	0.0506 (13)	
H8A	0.3336	1.1162	0.6749	0.061*	
H8B	0.4221	1.1062	0.5829	0.061*	
C9	0.2998 (5)	0.7295 (7)	0.7150 (4)	0.0421 (11)	
C10	0.2338 (5)	0.5493 (7)	0.6755 (4)	0.0494 (12)	
H10	0.2479	0.4934	0.6087	0.059*	
C11	0.1475 (5)	0.4496 (7)	0.7317 (4)	0.0528 (13)	
H11	0.1046	0.3277	0.7042	0.063*	
C12	0.1266 (5)	0.5352 (8)	0.8298 (4)	0.0498 (12)	
C13	0.1879 (6)	0.7160 (9)	0.8705 (5)	0.0616 (15)	
H13	0.1721	0.7721	0.9367	0.074*	
C14	0.2725 (6)	0.8132 (7)	0.8127 (4)	0.0548 (13)	
H14	0.3124	0.9373	0.8393	0.066*	

### Atomic displacement parameters (Å^2^)

**Table:**

	*U*^11^	*U*^22^	*U*^33^	*U*^12^	*U*^13^	*U*^23^
Br1	0.0668 (3)	0.1015 (5)	0.0779 (4)	−0.0184 (4)	0.0426 (3)	−0.0001 (4)
O1	0.077 (2)	0.091 (4)	0.0396 (18)	0.004 (2)	0.0162 (17)	0.010 (2)
O2	0.089 (3)	0.052 (3)	0.070 (3)	−0.007 (2)	0.018 (2)	−0.014 (2)
O3	0.073 (2)	0.0688 (19)	0.0500 (18)	−0.006 (3)	0.0329 (16)	0.001 (3)
O4	0.105 (3)	0.120 (5)	0.054 (2)	0.013 (3)	0.002 (2)	−0.020 (3)
O5	0.070 (3)	0.150 (5)	0.124 (4)	0.035 (3)	−0.005 (3)	−0.052 (4)
N1	0.070 (3)	0.059 (3)	0.059 (3)	0.004 (2)	0.008 (3)	−0.001 (2)
C1	0.051 (3)	0.039 (3)	0.043 (2)	0.002 (2)	0.020 (2)	0.001 (2)
C2	0.051 (3)	0.037 (2)	0.045 (2)	0.0014 (19)	0.023 (2)	0.0041 (19)
C3	0.050 (3)	0.052 (3)	0.067 (3)	0.005 (2)	0.024 (3)	0.012 (3)
C4	0.081 (5)	0.073 (4)	0.073 (4)	0.012 (3)	0.024 (4)	0.029 (4)
C5	0.048 (3)	0.065 (4)	0.046 (3)	−0.003 (2)	0.016 (2)	0.003 (2)
C6	0.054 (3)	0.046 (2)	0.057 (3)	−0.003 (3)	0.025 (2)	0.001 (3)
C7	0.062 (3)	0.119 (7)	0.079 (4)	−0.024 (4)	0.028 (3)	0.001 (4)
C8	0.055 (3)	0.042 (3)	0.052 (3)	0.003 (3)	0.010 (3)	−0.001 (2)
C9	0.043 (3)	0.041 (2)	0.046 (3)	0.001 (2)	0.017 (2)	0.000 (2)
C10	0.057 (3)	0.048 (3)	0.049 (3)	−0.007 (2)	0.025 (2)	−0.012 (2)
C11	0.053 (3)	0.053 (3)	0.059 (3)	−0.003 (2)	0.027 (2)	−0.006 (2)
C12	0.039 (3)	0.064 (3)	0.050 (3)	−0.002 (2)	0.019 (2)	0.002 (3)
C13	0.054 (3)	0.083 (4)	0.056 (3)	−0.009 (3)	0.028 (3)	−0.020 (3)
C14	0.062 (3)	0.056 (3)	0.056 (3)	−0.013 (2)	0.033 (3)	−0.021 (2)

### Geometric parameters (Å, °)

**Table:**

Br1—C12	1.900 (5)	C4—H4A	0.9700
O1—C5	1.332 (6)	C4—H4B	0.9700
O1—C4	1.470 (8)	C6—C7	1.497 (7)
O2—C5	1.199 (6)	C7—H7A	0.9600
O3—C6	1.210 (5)	C7—H7B	0.9600
O4—N1	1.208 (6)	C7—H7C	0.9600
O5—N1	1.204 (6)	C8—H8A	0.9700
N1—C8	1.489 (7)	C8—H8B	0.9700
C1—C8	1.521 (6)	C9—C10	1.380 (6)
C1—C9	1.532 (6)	C9—C14	1.391 (6)
C1—C2	1.562 (7)	C10—C11	1.376 (7)
C1—H1	0.9800	C10—H10	0.9300
C2—C5	1.532 (6)	C11—C12	1.375 (7)
C2—C6	1.541 (6)	C11—H11	0.9300
C2—C3	1.555 (6)	C12—C13	1.371 (7)
C3—C4	1.522 (8)	C13—C14	1.369 (7)
C3—H3A	0.9700	C13—H13	0.9300
C3—H3B	0.9700	C14—H14	0.9300
			
C5—O1—C4	111.3 (4)	O3—C6—C2	120.0 (4)
O5—N1—O4	123.4 (5)	C7—C6—C2	118.1 (4)
O5—N1—C8	118.7 (5)	C6—C7—H7A	109.5
O4—N1—C8	117.9 (5)	C6—C7—H7B	109.5
C8—C1—C9	111.2 (4)	H7A—C7—H7B	109.5
C8—C1—C2	111.9 (4)	C6—C7—H7C	109.5
C9—C1—C2	113.0 (4)	H7A—C7—H7C	109.5
C8—C1—H1	106.8	H7B—C7—H7C	109.5
C9—C1—H1	106.8	N1—C8—C1	109.1 (4)
C2—C1—H1	106.8	N1—C8—H8A	109.9
C5—C2—C6	110.0 (4)	C1—C8—H8A	109.9
C5—C2—C3	103.3 (4)	N1—C8—H8B	109.9
C6—C2—C3	108.6 (4)	C1—C8—H8B	109.9
C5—C2—C1	111.7 (4)	H8A—C8—H8B	108.3
C6—C2—C1	110.9 (4)	C10—C9—C14	117.7 (4)
C3—C2—C1	112.1 (4)	C10—C9—C1	119.9 (4)
C4—C3—C2	103.8 (4)	C14—C9—C1	122.4 (4)
C4—C3—H3A	111.0	C11—C10—C9	122.1 (5)
C2—C3—H3A	111.0	C11—C10—H10	118.9
C4—C3—H3B	111.0	C9—C10—H10	118.9
C2—C3—H3B	111.0	C10—C11—C12	118.2 (5)
H3A—C3—H3B	109.0	C10—C11—H11	120.9
O1—C4—C3	106.8 (4)	C12—C11—H11	120.9
O1—C4—H4A	110.4	C13—C12—C11	121.4 (5)
C3—C4—H4A	110.4	C13—C12—Br1	119.5 (4)
O1—C4—H4B	110.4	C11—C12—Br1	119.0 (4)
C3—C4—H4B	110.4	C14—C13—C12	119.4 (5)
H4A—C4—H4B	108.6	C14—C13—H13	120.3
O2—C5—O1	122.5 (5)	C12—C13—H13	120.3
O2—C5—C2	126.2 (5)	C13—C14—C9	121.1 (5)
O1—C5—C2	111.3 (4)	C13—C14—H14	119.5
O3—C6—C7	121.8 (4)	C9—C14—H14	119.5
			
C8—C1—C2—C5	60.9 (5)	C5—C2—C6—C7	43.1 (7)
C9—C1—C2—C5	−65.5 (5)	C3—C2—C6—C7	−69.3 (7)
C8—C1—C2—C6	−62.2 (5)	C1—C2—C6—C7	167.1 (5)
C9—C1—C2—C6	171.4 (4)	O5—N1—C8—C1	117.9 (6)
C8—C1—C2—C3	176.3 (4)	O4—N1—C8—C1	−62.9 (7)
C9—C1—C2—C3	49.8 (5)	C9—C1—C8—N1	−68.4 (5)
C5—C2—C3—C4	17.2 (5)	C2—C1—C8—N1	164.2 (4)
C6—C2—C3—C4	133.9 (5)	C8—C1—C9—C10	121.9 (5)
C1—C2—C3—C4	−103.2 (5)	C2—C1—C9—C10	−111.2 (5)
C5—O1—C4—C3	12.5 (7)	C8—C1—C9—C14	−59.0 (6)
C2—C3—C4—O1	−18.2 (6)	C2—C1—C9—C14	67.8 (6)
C4—O1—C5—O2	177.8 (5)	C14—C9—C10—C11	−2.3 (7)
C4—O1—C5—C2	−0.8 (6)	C1—C9—C10—C11	176.8 (5)
C6—C2—C5—O2	54.9 (7)	C9—C10—C11—C12	0.6 (8)
C3—C2—C5—O2	170.7 (5)	C10—C11—C12—C13	0.8 (8)
C1—C2—C5—O2	−68.7 (7)	C10—C11—C12—Br1	−177.7 (4)
C6—C2—C5—O1	−126.5 (4)	C11—C12—C13—C14	−0.4 (8)
C3—C2—C5—O1	−10.8 (5)	Br1—C12—C13—C14	178.1 (4)
C1—C2—C5—O1	109.9 (4)	C12—C13—C14—C9	−1.4 (9)
C5—C2—C6—O3	−140.6 (6)	C10—C9—C14—C13	2.7 (8)
C3—C2—C6—O3	107.0 (7)	C1—C9—C14—C13	−176.4 (5)
C1—C2—C6—O3	−16.5 (8)		

### Hydrogen-bond geometry (Å, °)

**Table:**

*D*—H···*A*	*D*—H	H···*A*	*D*···*A*	*D*—H···*A*
C11—H11···O5^i^	0.93	2.58	3.306 (2)	135
C10—H10···O3^ii^	0.93	2.63	3.514 (2)	158

Symmetry codes: (i) *x*, *y*−1, *z*; (ii) −*x*+1, *y*−1/2, −*z*+1.
